# *In vivo* comparison of mesh fixation solutions in open and laparoscopic procedures for inguinal hernia repair: A meta-analysis

**DOI:** 10.1016/j.heliyon.2024.e28711

**Published:** 2024-03-23

**Authors:** Cristiana Giordano, Elisabetta Rosellini, Maria Grazia Cascone, Francesca Di Puccio

**Affiliations:** Department of Civil and Industrial Engineering, University of Pisa, Largo Lucio Lazzarino, 56122, Pisa, Italy

**Keywords:** Abdominal hernia repair, Mesh fixation, Cyanoacrylate-based glue, Fibrin glue, Suture, Tack

## Abstract

**Background:**

Abdominal hernia repair surgeries involve the fixation of a surgical mesh to the abdominal wall with different means such as suture, tacks, and glues. Currently, the most effective mesh fixation system is still debated. This review compares outcomes of mesh fixation in different surgical procedures, aiding surgeons in identifying the optimal technique.

**Methods:**

A meta-analysis was conducted according to PRISMA guidelines. Articles published between January 2003 and January 2023 were searched in electronic databases. Randomized controlled trials (RCTs) comparing mesh fixation with cyanoacrylate-based or fibrin glues with classical fixation techniques (sutures, tacks) in open and laparoscopic procedures were included.

**Results:**

17 RCTs were identified; the cumulative study population included 3919 patients and a total of 3976 inguinal hernias. Cyanoacrylate-based and fibrin glues were used in 1639 different defects, suture and tacks in 1912 defects, self-gripping mesh in 404 cases, and no mesh fixation in 21 defects. Glue fixation resulted in lower early postoperative pain, and chronic pain occurred less frequently. The incidence of hematoma was lower with glue fixation than with mechanical fixation. Recurrence rate, seroma formation, operative and hospitalization time showed no significant differences; but significantly, a higher number of people in the glue group returned to work by 15- and 30-days after surgery when compared to the tacker and suture groups in the same time frame.

**Conclusion:**

Cyanoacrylate and fibrin glue may be effective in reducing early and chronic pain and hematoma incidence without increasing the recurrence rate, the seroma formation, or the operative and hospitalization time.

## 1 Introduction

Abdominal hernia is a very common clinical pathology that affects 4% of the population over 45 years and 1.7% at all ages. Two main categories of abdominal hernias are identified, primary and incisional. Although incisional and primary ventral hernias are frequently categorized together, it is crucial to recognize that each has a unique pathogenesis and varying patient risk factors. Consequently, distinct therapeutic strategies are often required for each type of hernia. Primary hernias are classified according to position and size, as estimated by the radius of the defect. Consequently, epigastric, umbilical, spigelian and lumbar hernias are distinguished, and, according to diameter, they are divided into small (<2 cm), medium (2–4 cm) and large (>4 cm) hernias. Incisional hernias, are also classified according to position and size [1,2]. Regarding the location of the hernia, the abdomen is divided into a medial and a lateral zone and, for these two regions can be identified the following hernia types: subxiphoidal, epigastric, umbilical, infraumbilical and suprapubic, for the medial zone instead for the latera zone can be identified the subcostal, the flank, the iliac and the lumbar hernia [2]. An important aspect to evaluate is that about 75% of abdominal hernias are inguinal and occur mainly in men, due to the presence of weaknesses at the groin level [3,4]. Approximately twenty million repair surgeries of inguinal hernia are performed each year worldwide. These numbers highlight the impact of this problem and of the outcomes of surgical procedures on the healthcare system. The most reliable and safe solution to repair these defects is still debated. A variety of surgical techniques are performed for both open (e.g. Lichtenstein, Trabucco, Rives-Stoppa), laparoscopic (e.g. Transabdominal pre‐peritoneal - TAPP, totally extraperitoneal - TEP, intraperitoneal onlay mesh - IPOM), and robotic procedures (e.g. Robotic-Assisted Transabdominal Preperitoneal Repair - r-TAPP, Transversus abdominis release procedure – TAR, and Robotic Transabdominal Retromuscular Umbilical Prosthetic Hernia Repair – TARUP).

Traditional hernia repair involves open surgery (OS) with a larger incision, allowing direct access to the hernia. This approach is beneficial for complex or recurrent hernias but requires a longer recovery and it is associated with a higher risk of complications like infections. In contrast, laparoscopic surgery (LS) is minimally invasive, utilizing small incisions, it can offer the advantages of reduced pain, quicker recovery, and lower complication risks [5]. Robotic surgery (RS) combines advantages of open and laparoscopic methods, offering enhanced visualization and precision. However, the high cost of robotic systems and the need for specialized training can limit its availability and use. Moreover, further research is needed to fully understand the long-term outcomes of laparoscopic and robotic surgery. The choice between open, laparoscopic, and robotic surgery for abdominal hernia repair is multifactorial. It depends on the specifics of the hernia, the health status of patients, the expertise of the surgeon and the available resources [6,7].

In all the surgical approaches, the most common type of surgical repair uses a mesh to close the defect [8]. It reduces recurrence rates by 2.7% versus 8.2% in ventral hernia repair, and by 50–75% in cases of inguinal hernia repair, compared with hernia reconstruction using no-mesh method [9,10]. Different kind of meshes are available for abdominal hernia repair: synthetic, biological and hybrid. The synthetic meshes are made from material like polypropylene, polytetrafluoroethylene, polyurethane; they are durable and provide strength to the damage tissue, promoting faster healing. Biological meshes are derived from human, bovine and porcine decellularized tissue, they are designed to degrade over time while supporting the repair site. By combining the distinct advantages and drawbacks of both biologic and synthetic materials, it may be feasible to create a hybrid approach that maximizes the benefits of each while minimizing their respective drawbacks [11]. Other important aspects to consider are the porosity, weight and monofilament or multifilament structure of the prosthesis. The selection of mesh can impact the surgical procedure significantly. Prior to choosing a mesh for a specific patient, the surgeon needs to consider various patient characteristics (such as age, defect size, obesity, underlying disease process, etc.) and mesh properties to determine the most suitable treatment. Nowadays, synthetic meshes, specifically polypropylene meshes, are the most widely employed.

Ensuring mechanical compatibility between the hernia meshes and the layers of the abdominal wall is crucial in preventing postoperative complications and recurrences [12]. The use of mesh generally requires fixation elements such as suture, glues or tacks, making exception self-gripping meshes that do not require mechanical fixation but adhere to tissues. The major limitation of self-gripping meshes is related to the defect size. Due to difficulties in producing meshes wider than 15 cm with adequate homogeneity, these prostheses are not suitable for large defects [13]. Moreover, from the mechanical point of view, mesh fixation gives superior results compared to the nonfixed solutions, in term of mesh motion and tensile strength [14]. However, also mesh fixation may have adverse outcomes, the main one being chronic pain, which occurs in approximately 10–40 % of patients after surgery [15], probably due to nerve injury or tissue damage. Nonetheless, sutures and tacks are considered the gold standard means for fixing meshes. In the last two decades, adhesive biomaterials have demonstrated similar or even better postoperative results. Among tissue adhesives, fibrin and cyanoacrylate-based glues are the most used for mesh fixation in abdominal hernia repair procedures [16]. It should be noted, however, that both solutions present some limitations. Different formulations of fibrin glue are available, which have different concentration of fibrinogen and thrombin; the tensile strength of this kind of glue depends on fibrinogen concentration, which range from 40 to 115 mg/ml [17,18]. The adhesive strength of fibrin glues results lower if compared to cyanoacrylate-based glues, i.e. 64.3 N vs. 105.4 N according in an *in vitro* study [19]. In addition, fibrin glues are haemostatic agents rather than adhesives, compared with cyanoacrylate-based glues, which are instead categorized as sealants, adhesives and haemostatic agents [20]. N-butyl-cyanoacrylate and n-hexyl cyanoacrylate, however, can have a apoptotic effect, which was originally attributed to heat release during the exothermic polymerization process [16]. This limitation has been overcome by new surgical glues composed of N-butyl-2-cyanoacrylate (n-BCA) and methacryloxysulfolane monomer (MS). As a result of the longer radical chain, a lower polymerization temperature is achieved, resulting in lower toxicity and fewer inflammatory reactions [21]. When used as mesh fixation devices, both cyanoacrylate-based and fibrin glues seem to reduce postoperative complications, such as pain and hematoma formation. Moreover, they can be used in very critical and sensitive areas like the triangles of doom and pain and the diaphragm area [22]. Considering the important structures located in these areas it has been strongly recommended to avoid the traumatic fixation of mesh as it could cause major vascular or nerve injury that could result in chronic pain [15]. The diaphragm area shows strenuous requirements as well, due to the proximity to vital organs such as heart and lungs. Fixation with tacks and suture may be feasible but it should generally be avoided to prevent lung or cardiac injury or injuries to the neurovascular bundles running along the inferior surface of each rib [23]. In alternative, in recent years, n-butyl-2-cyanoacrylate, n-hexyl cyanoacrylate and fibrin glue have been identified as suitable for mesh fixation in abdominal hernioplasty and several randomized controlled trials have compared mesh fixation with suture and tissue adhesives [24]. This meta-analysis aims to summarize the evidence of current studies on the benefits, risks, and limitations of existing mesh fixation techniques for abdominal hernia repair, in different surgical procedures. The use of tacks, tissue adhesives and suture as methods of mesh fixation were compared in terms of early and chronic pain, hernia recurrence, seroma and hematoma formation, hospitalization and operative time [25]. The effectiveness of invasive and non-invasive fixation systems is evaluated in inguinal hernia repair procedures. A double analysis is conducted to distinguish data from open or laparoscopic surgery in RCTs using synthetic prostheses, this reduces the chance of variability caused by surgical procedures.

## 2 Materials and methods

### 2.1 Search strategy

**Fig. 1:**
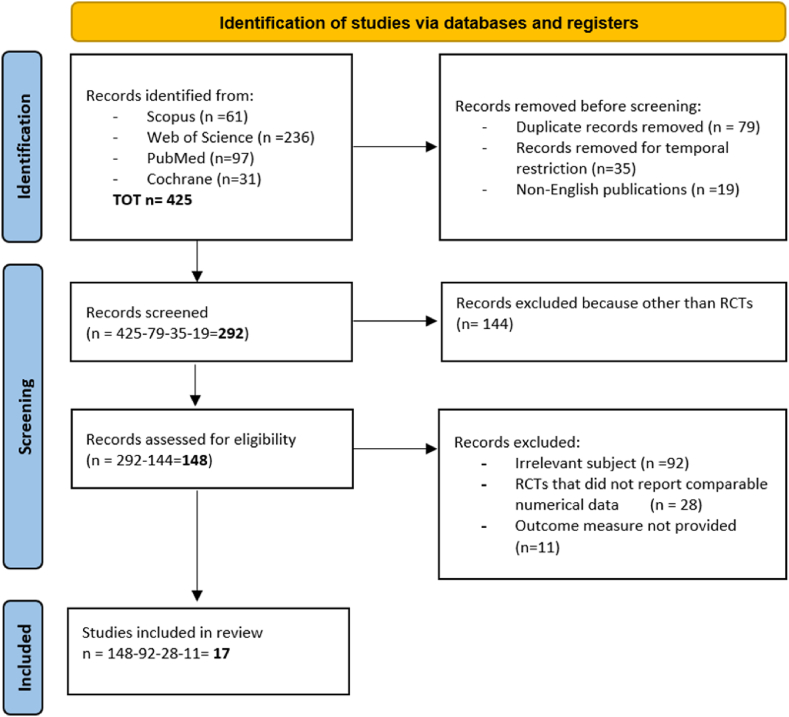
PRISMA 2020 flow diagram for new systematic reviews which included searches of databases only. 17 randomized clinical trials (RCT) were considered at the end of the screening process.

A Meta-analysis was performed according to the PRISMA guidelines using the following databases: Scopus, Web of Science, PubMed and Cochrane. The last update of the research was January 20, 2023. As keywords “abdominal hernia repair”, “mesh fixation”, “cyanoacrylate-based glue”, “fibrin glue”, “suture”, “tack” were used and combined with the Boolean connectors “AND” and “OR”. The search was restricted to article titles, keywords and abstracts, in research papers published between 2003 and 2023, in English, and linked with abdominal surgery and clinical trials. [Example of search algorithms or key terms for Scopus: (TITLE-ABS-KEY(Abdominal hernia repair AND mesh fixation AND glue AND suture OR tack) AND PUBYEAR >2003 AND PUBYEAR <2023 AND (LIMIT-TO (DOCTYPE,"ar")) AND (LIMIT-TO (SUBJAREA,"MEDI")) AND (LIMIT-TO (LANGUAGE,"English")))]. All titles were initially evaluated, relevant abstracts were extracted, and duplicates were excluded. Studies with incomplete information, which did not provide numerical data and postoperative outcomes, were excluded from the analysis. Selected studies include evaluations of adult patients subjected to abdominal, in particular inguinal, hernia repair surgery. The PRISMA flow chart summarising the study selection process is shown in Fig. 1.

### 2.2 Data extraction

The initial search resulted in 425 articles, after the removal of 79 duplicates, 19 non-English papers and 35 articles published before 2003, 292 results were evaluated for eligibility. All 292 titles were initially examined, and 148 relevant abstracts connected with abdominal surgery and randomized clinical trials on adult patients were extracted. These potentially relevant articles were evaluated but not all of them presented relevant and comparable data so in the end only 17 were selected and included in the following meta-analysis. Articles that seemed potentially relevant were retrieved and analysed by the reviewers and the main data were extracted from each article. The following data were retrieved: first author, publication year, study period, number of patients and their age, male vs female ratio, number and type of hernia, type of surgical and fixation techniques, type of mesh used and follow up time. In particular, the outcomes of the surgical procedures were classified in three categories: primary, secondary and additional outcomes.

The primary outcomes of interest are:

a) Early pain; it is evaluated with the Visual Analogue Scale (VAS) and with questionnaires useful to understand the patient discomfort after surgery procedure [26]. The presence of early pain requires analgesics and is also associated with a longer hospital stay which may have socio-economic implications [27]
b) Chronic pain; pain becomes chronic when persists for three months after surgery with VAS index higher or equal to 4 cm and it is the main factor causing poor patient satisfaction after abdominal hernia repair [28]
c) Hernia recurrence at the end of the follow-up period. The presence of a recurrence requires a second intervention resulting in patient discomfort and health care costs

Secondary outcomes are:

d) Seroma
e) Hematoma

Other important outcomes evaluated in this meta-analysis are:

f) The duration of surgery
g) The hospital stay
h) The assessment of the return to normal activity of patients.

Data were analysed to assess differences between mesh fixation methods in inguinal hernia repair procedures. The statistical analysis was differentiated according to the surgical approach used between open and laparoscopic techniques. To avoid that different mesh materials influenced the analysis, only studies in which polypropylene meshes were used were evaluated. As data on comparative effectiveness and adverse events were reported heterogeneously in the studies, we did not standardise the numerical results.

### 2.3 Statistical analysis

The statistical analysis was conducted using MedCalc statistic software and data were compared using the Odd Ratio (OR). The 95% confidence interval (CI) was reported for each analysed value. The studies without useful numerical data were not included in the statistical analysis. For a graphical representation of the results, we used Forest plots; each outcome is represented on a graph by a square dot, with horizontal line extensions representing the 95 % CIs. The different sizes of the squares are indicators of the different weight of the study in the overall statistics. A diamond shape represents the overall effect, its width represents the 95 % CIs. The vertical line in the graph intersecting 1 is the line of no effect. If the horizontal lines cross the no effect line, then no one of mesh fixation method was considered significant. Heterogeneity was assessed using the I^2^ statistic. Publication bias was assessed visually evaluating the symmetry of funnel plots (Supplementary material); additionally, Egger's test was applied.

## 3 Results

### 3.1 Overview of selected studies

The initial research analysed papers of the last two decades, but the selected articles were published between 2006 and 2022. The cumulative study population includes 3919 patients subjected to surgical hernia repair and, evaluating unilateral and bilateral defects, there are 3976 repaired hernias. The number of participants in the different studies ranges from 24 to 625 and they are all adults aged 18–85 years old. Ten papers out of 17 considered both male and female patients with a prevalence of men [21,25,[29], [30], [31], [32], [33], [34], [35]], other six considered only male patients [[36], [37], [38], [39], [40], [41]] and in one paper the gender of the patients was not reported [42]. Eleven of the analysed papers compared different mesh fixation methods in open hernia repair [21,30,[34], [35], [36], [37], [38], [39], [40],^42^,^43^], five evaluated laparoscopic procedures [25,29,31,32,41] and one RCT analysed both open and laparoscopic procedures [33]. Mesh fixation means included in these studies are cyanoacrylate glue, fibrin glue, sutures, tacks, and self-gripping meshes. Cyanoacrylate-based glues and fibrin glues were used in 1639 different defects in particular 867 meshes were fixed with cyanoacrylate-based glue and 772 with fibrin glue; suture and tacks were used in 1912 defects in particular 1210 meshes were fixed with suture and 702 with tacks. 404 self-gripping meshes were also used and the no mesh fixation method was used only in 21 defects [29,30]. All selected articles examined the use of synthetic prostheses in inguinal hernia repair procedures. Summary of study characteristics is reported in Table 1.

**Table 1:** Study characteristics of the included 17 papers.

First author	Publication year	Study period	Number of patients	Age, years (range)	M:F ratio	*Number and* Type of inguinal hernia	Type of surgical technique		Type of mesh	Follow up duration
							Open or Laparoscopic	Fixation technique		
Yassin [34]	2022	NR	24	20–60	24:0	Tot: 24 primary Direct (5) Indirect (19)	Open	Cyanoacrylate Glue (12), sutures (12)	Polypropylene	6 months
Zaidan [35]	2022	May 2016–March 2017	60	21–55	60:0	Tot: 60 Primary unilateral (60)	Open -Lichtenstein	Cyanoacrylate Glue (30), sutures (30)	Polypropylene	6 months
Azevedo [44]	2022	November 2016–November 2019	63	18–75	61:2	Tot: 63 Primary unilateral (63)	Laparoscopic- TAPP	N-butyl 2 cyanoacrylate and methacryloxy sulfolane glue (21), tackers (21), non-fixation (21)	heavyweight polypropylene	24 months
Matikainen [45]	2020	January 2012–December 2013	625	>18	605:20	Tot: 634 primary unilateral and bilateral (610) recurrent (24)	Open -Lichtenstein	cyanoacrylate glue (216), self-gripping mesh (202) or nonabsorbable 3–0 polypropylene sutures (216)	lightweight polypropylene	60 months
Matikainen [29]	2018	January 2012–December 2013	625	>18	585:40	Tot: 625 Primary (597) Recurrent (28)	Open	Cyanoacrylate Glue (216), non-absorbable sutures (207) self-gripping mesh (202)	polypropylene self-gripping mesh	24 months
Chandra [46]	2016	October 2012–November 2014	101	18–60	73:27	Tot: 101 Unilateral: Direct (69) indirect (24) Pantaloon (8)	Laparoscopic-TEP	Tacker (50) Fibrin glue (50)	NR	3 months
Damiano [40]	2014	January 2004–February 2010	468	>18	NR	Tot: 468 Primary unilateral (468)	Open -Lichtenstein	Polypropylene suture (252), Fibrin glue (216)	NR	12 months
Moreno-Egea [31]	2014	January 2008–January 2011	208	>18	71:31	Tot: 208 Primary(208)	Open-Lichtenstein (102) TEP (106)	Prolene sutures (52), Cyanoacrylate glue 102), Tackers (54)	Lightweight polypropylene-coated titanium	12 months
Mikhail [32]	2012	September 2009–May 2011	198	20–85	169:29	Tot: 198 Primary (198)	Open	N-butyl 2 cyanoacrylate and methacryloxy sulfolane glue (101), sutures (98)	NR	12 months
Brügger [25]	2012	NR	80	19–82	79:1	Tot: 80 Primary (80)	Laparoscopic-TAPP	Spiral tacks (40), N-butyl 2 cyanoacrylate and methacryloxy sulfolane (40)	Lightweight multifilament mesh	Average of 38 months
Campanelli [36]	2012	January 2006–April 2007	319	18–80	319:0	Tot: 319 Primary (319)	Open -Lichtenstein	Fibrin glue (159), Traditional suture (160)	Heavyweight polypropylene	12 months
Shen [33]	2012	January 2010–April 2010	110	40–78	92:18	Tot: 110 Primary unilateral (110) direct (14), indirect (96)	Open -Lichtenstein	n-butyl-2-cyanoacrylate glue (55), suture (55)	Lightweight polypropylene	15 months
Sözen [37]	2012	January 2009–July 2009	116	20–75	116:0	Tot: 116 Primary direct (46) indirect(64) combined(6)	Open -Lichtenstein	Fibrin glue (62), Polypropylene suture (54)	polypropylene	12 months
Dąbrowiecki [38]	2012	July 2008–November 2010	41	>21	41:0	Tot: 41 Primary (41)	Open -Lichtenstein	N-butyl 2 cyanoacrylate and methacryl-oxy-sulfolane glue (20), 2-0 polypropylene suture (21)	Polypropylene mesh	12 months
Testini [21]	2010	January 2003–December 2007	156	17–85	144:12	Tot: 167 Primary Direct(76) Indirect (71) Combine (9)	Open	Suture (53), fibrin glue (49) and N-butyl-2-cyanoacrylate (54)	polypropylene mesh and plug	12 months
Olmi [43]	2007	September 2001–September 2004.	600	<80	588:12	Tot: 803 Primary (707) Recurrence (96)	Laparoscopic - TAPP	Mechanical system (450), fibrin glue (150)	Polypropylene	1 month
Schwab [39]	2006	2002–2004	125	20–80	125:0	Tot: 173 Primary (173)	Laparoscopic-TEP	Staples (87), fibrin glue (86)	NR	Average of 23.7 months

NR = not reported.

### 3.2 Primary outcomes

#### 3.2.1 Early pain

**Fig. 2:**
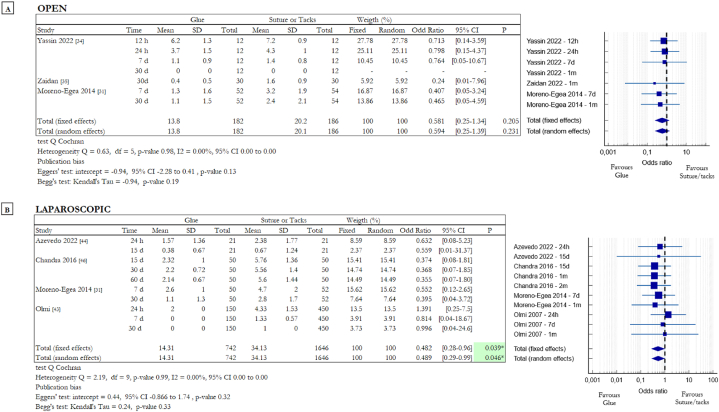
Summary data and forest plots comparing VAS score in different moments following glue fixation versus suture or tack fixation of mesh in inguinal hernia repair procedures. In the Time column, h stands for hours and d stands for days. The Mean column includes the mean values of the VAS scores, instead the SD column includes the standard deviation values of the scores. Odd ratios are charted with 95% confidence intervals. **A** Data obtained from open surgery (OS). **B** Data obtained from laparoscopic surgery (LS).

Early and chronic pain are elements of interest to evaluate the effects of different mesh fixation techniques. All the chosen papers analysed these aspects, but in particular 3 reported data related to early pain in OS [31,34,35] and 4 related to early pain in LS [31,43,44,46], 8 studies reported data related to chronic pain in OS [21,[31], [32], [33],[35], [36], [37],^45^] and 5 reported data on chronic pain in LS [25,31,39,43,46]. Patients were asked to record pain scores 12 or 24 h after the surgical procedure using the VAS index. The same procedure was repeated after 7, 15, 30 and maximum 60 days. In this way it is possible to obtain data on the perception of early pain as we can see in Fig. 2. Evaluating the data of different clinical trials in different moments after surgery and in different surgical approaches, we obtained the Forest plots with the ORs obtained analysing the mean values of the VAS scores. Moreno-Egea et al. compared glue with both suture and tacks in OS and LS, for this reason, this study appears in the tables in Fig. 2A and B [31]. The statistical analysis of data related to OS gave the following result: OR 0.594, 95 % CI 0.25 to 1.39 (random effects model) with a p-value of 0.23. There was no heterogeneity among the studies (Q = 0.63, df = 5, p = 0.99, I2 = 0 %). The result shows a less pain perception in patients in the glue group but not in a statistically significant way. For LS the following results were obtained: OR 0.489, 95 % CI 0.29 to 0.99 (random effects model) with a p-value of 0.046*. There was no heterogeneity among the studies (Q = 2.19, df = 9, p = 0.99, I2 = 0 %). The results in this case show a statistically significant difference between the data in favour of the glue group. The use of tissue adhesive as mesh fixation method, in this case, results in a significant lower early pain compared with invasive methods highlighting the benefits of a non-traumatic procedure.

#### 3.2.2 Chronic pain

**Fig. 3:**
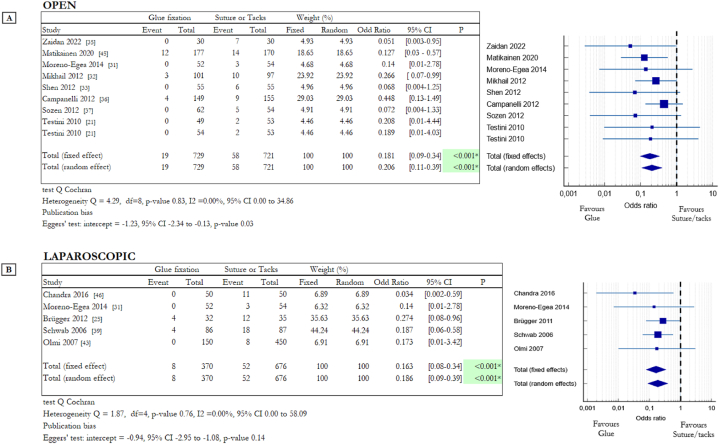
Summary data and forest plot comparing chronic pain following glue fixation versus suture or tack fixation of mesh in inguinal hernia repair procedures. Event represents the number of patients with chronic pain after three or more months out of the total number of patients involved in the study. Odd ratios are charted with 95% confidence intervals. **A** Data obtained from open surgery (OS). **B** Data obtained from laparoscopic surgery (LS).

Chronic pain is a condition that occurs when pain persists for more than three months with a VAS index greater than or equal to 4. This is one of the most significant aspects to assess in relation to abdominal hernia repair procedures. Fig. 3 shows the result for chronic pain and the numbers of patients with this problem three or more months after the surgery procedure (open: Fig. 3A, laparoscopic: Fig. 3B). For the open approach six RCTs compared cyanoacrylate-based glue with suture [21,[31], [32], [33],^35^,^45^] and three compared fibrin glue with suture [21,36,37]. Testini et al. compared suture with both fibrin and cyanoacrylate-based glues, for this reason, this study appears twice in the table in Fig. 3A [21].

In laparoscopic surgery two RCTs compared cyanoacrylate-based glue with tacks [25,31] instead three evaluated the differences between fibrin glue and tacks [39,43,46]. Moreno-Egea et al. considered data obtained from OS and LS, for this reason, this study appears in tables in Fig. 3A and B. As highlighted in Fig. 3 in many cases there is a significant difference between the use of invasive and non-invasive systems.

Analysing the collected dataset on OS, it results that in the suture/tacks group 58 of 721 patients suffer chronic pain, instead only 19 of 729 patients of the glue group have the same discomfort (8.04% vs 2.61%). The difference between the two groups is statistically significant in favour of glues: OR 0.206, 95% CI 0.11 to 0.39 (random effects model) with a p-value <0.001* (heterogeneity Q = 4.29, df = 8, p = 0.83, I2 = 0.0 %). Even in LS, there is a statistically significant difference between penetrative and non-penetrative fixation systems. In the suture/tack group 52 of 676 patients suffer chronic pain, instead of 8 of 370 in the glue group (7.69% vs 2.13%). The difference between the two groups is statistically significant: OR 0.186, 95% CI 0.09 to 0.39 (random effects model) with a p-value <0.001* (heterogeneity Q = 1.87, df = 4, p = 0.76, I2 = 0.0 %). Atraumatic mesh fixation methods significantly reduce the incidence of chronic pain that is, instead, a relevant problem connected with penetrative systems like suture or tacks.

#### 3.2.3 Hernia recurrence

**Fig. 4:**
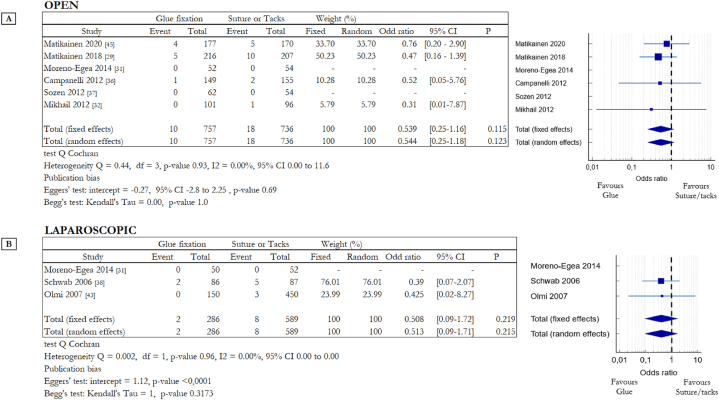
Summary data and forest plot comparing the rate of hernia recurrence following glue fixation versus suture or tack fixation of mesh in inguinal hernia repair procedures. Event represents the number of patients with hernia recurrence after twelve or more months out of the total number of patients involved in the study. Odd ratios are charted with 95% confidence intervals. **A** Data obtained from open surgery (OS). **B** Data obtained from laparoscopic surgery (LS).

The analysis of data focused in hernia recurrence is summarized in Fig. 4. In most of the RCTs, the time for assessment of recurrence was not clearly reported; when reported, the incidence of recurrence was assessed after a follow-up period of 12 months or more. The incidence of hernia recurrence was evaluated analysing different kinds of mesh fixation techniques. Among the studies on OS, 4 studies compared cyanoacrylate-based glue with suture [29,31,32,45] and 2 fibrin glue with suture [36,37]. In LS one RCT compared cyanoacrylate-based glue with tacks [31] and two studies evaluated the different impact of fibrin glue and tacks [39,43]. Moreno-Egea et al. considered data obtained from open and laparoscopic procedures, for this reason, this study appears in tables in Fig. 4A and B. The Forest plot in Fig. 4A compares all the data from six studies related to OS; there was no heterogeneity among the studies (Q = 0.44, df = 3, p = 0.69, I2 = 0 %). Recurrence occurred in 10 out of 757 patients of the non-invasive solution group, versus 18 out of 736 of the invasive one (1.32% vs 2.44%). Data are slightly in favour of surgical glues but not in a statistically significant way: OR 0.544, 95% CI 0.25 to 1.18 (random effects model) with a p-value = 0.123. The Forest plot in Fig. 4B instead compares all the data from three studies of LS; in this case recurrence occurred in 2 out of 286 patients of the non-invasive solution group, versus 8 out of 589 of the invasive one (0.7% vs 1.36%). Data are slightly in favour of surgical glues but, also in laparoscopic surgery, not in a statistically significant way: OR 0.513, 95% CI 0.09 to 1.71 (random effects model) with a p-value = 0.215 (heterogeneity Q = 0.002, df = 1, p = 0.96, I2 = 0.0 %).

### 3.3 Secondary outcomes

#### 3.3.1 Seroma

**Fig. 5:**
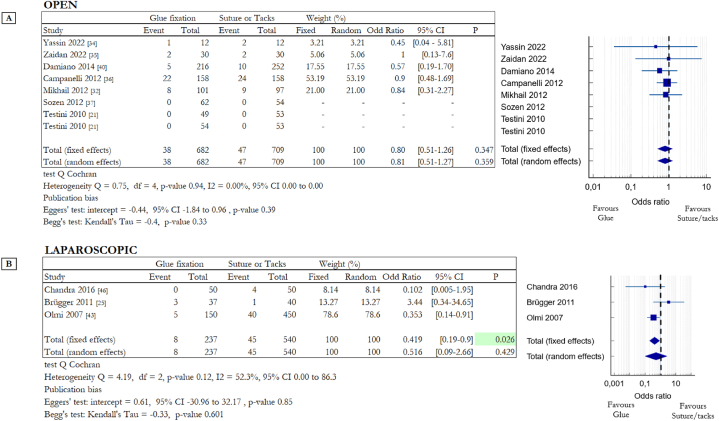
Summary data and forest plot comparing seroma formation following glue fixation versus suture or tack fixation of mesh during inguinal hernia repair procedures. Event represents the number of patients having seroma formation after surgery out of the total number of patients involved in the study. Odd ratios are charted with 95% confidence intervals. **A** Data obtained from open surgery (OS). **B** Data obtained from laparoscopic surgery (LS).

Eight studies underwent meta-analysis for seroma formation in open surgery. Four RCTs evaluated seroma formation in suture and cyanoacrylate-based glue [21,32,34,35], four studies compared the use of suture and fibrin glue [21,36,37,40]. Testini et al. compared suture with both fibrin and cyanoacrylate-based glue, for this reason, this study is reported twice in the table in Fig. 5A [21]. For laparoscopic approach three studies were analysed, two on fibrin glue and tacks [43,46] and one on the comparison between cyanoacrylate-based glue and tacks [25]. Fig. 5 shows the Forest plots for seroma formation with glue, suture or tacks fixation of mesh in inguinal hernia repair procedures. From Fig. 5A (open surgery) the following results are obtained: OR 0.81, 95% CI 0.51 to 1.27(random effects model) with p-value 0.359, (heterogeneity Q = 0.75, df = 4, p = 0.94, I2 = 0.0 %). From laparoscopic procedures the results are: OR 0.516, 95% CI 0.09 to 2.66 (random effects model) with p-value 0.429, (heterogeneity Q = 4.19, df = 2, p = 0.12, I2 = 0.0 %). In both surgical procedures, the data are only slightly in favour of surgical glues, but no statistically significant difference appears between the use of invasive and non-invasive fixation systems.

#### 3.3.2 Hematoma

**Fig. 6:**
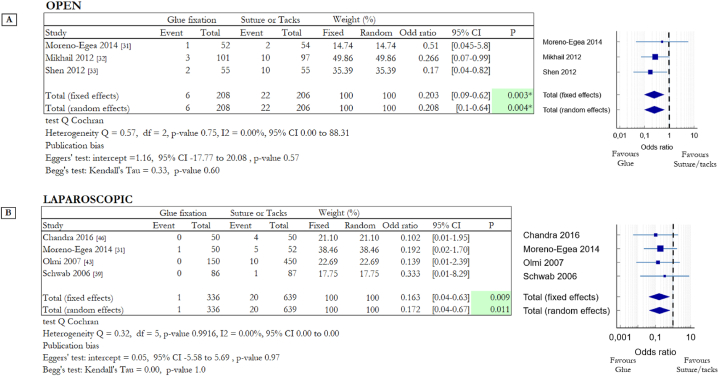
Summary data and forest plot comparing hematoma formation following glue fixation versus suture or tack fixation of mesh during inguinal hernia repair procedures. Event represents the number of patients with hematoma formation after surgery out of the total number of patients involved in the study. Odd ratios are charted with 95% confidence intervals. **A** Data obtained from open surgery (OS). **B** Data obtained from laparoscopic surgery (LS).

Hematoma incidence after inguinal hernia surgery was evaluated in three different studies for OS and in four studies for LS. In the first case all the three RCTs evaluated the differences between cyanoacrylate-based glue and suture [[31], [32], [33]] instead, in laparoscopic approach one study analysed cyanoacrylate-based glue and tacks [31], the others evaluated fibrin glue and tacks [39,43,46]. Moreno-Egea et al. considered data obtained from OS and LS, for this reason, this study appears in tables in Fig. 6A and B. Analysing all the data in Fig. 6A only 2.88% of patients in the glue group had hematoma formation in contrast with 10.68% in the suture/tacks group. Analysing the Forest plot obtained from the evaluation of the ORs, it can be seen a statistically significant difference between the groups with all data in favour of glue and with the following results: OR 0.208, 95% CI 0.1 to 0.64, p-value 0.004*(random effects model; heterogeneity Q = 0.57, df = 2, p = 0.75, I2 = 0 %). From laparoscopic procedures, 0.3% of patients in the glue group had hematoma formation in contrast with 3.13% in the suture/tacks group and, from the table in Fig. 6B the following results are obtained: OR 0.172, 95% CI 0.04 to 0.67, p-value 0.011*(random effects model; heterogeneity Q = 0.32, df = 5, p = 0.99, I2 = 0 %).

### 3.4 Additional outcomes

#### 3.4.1 Operative time

**Fig. 7:**
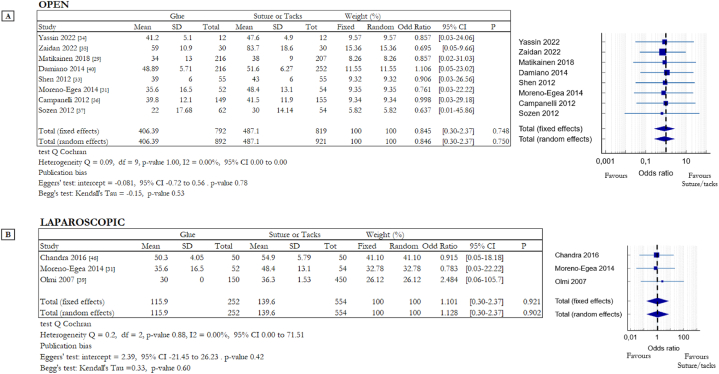
Summary data and forest plot comparing time required for the surgical procedure in minutes following glue fixation versus suture or tack fixation of mesh during inguinal hernia repair. The Mean column includes the mean values of time required for surgery, instead the SD column includes the standard deviation of these values. Odd ratios are charted with 95% confidence intervals. **A** Data obtained from open surgery (OS). **B** Data obtained from laparoscopic surgery (LS).

Another important aspect to consider is the time required for surgery. Data extracted from studies on open and laparoscopic surgery were analysed to assess whether the use of adhesives or traditional mesh fixation systems affected the time required for the surgical procedure. Of the 8 studies on OS, 5 analysed the difference between cyanoacrylate-based glue and suture [31,[33], [34], [35],^45^] while the other 3 focused on fibrin glue and suture [36,37,40]. Of the three studies on LS, two compared tacks and fibrin glue [43,46] and one compared tacks and cyanoacrylate-based glue [31]. Moreno-Egea et al. considered data obtained from open and laparoscopic procedures, for this reason, this study appears in tables in Fig. 7A and B. From the analysed studies, no difference between invasive and non-invasive fixation systems was obtained, there was no difference in time required for the surgical procedure between the two groups. The results of the statistical analysis are: OR 0.846, 95% CI 0.30 to 2.37, p-value 0.75, (random effects model) in OS and OR 1.13, 95% CI 0.30 to 2.37, p-value 0.90, (random effects model) in LS.

#### 3.4.2 Hospitalization time

**Fig. 8:**
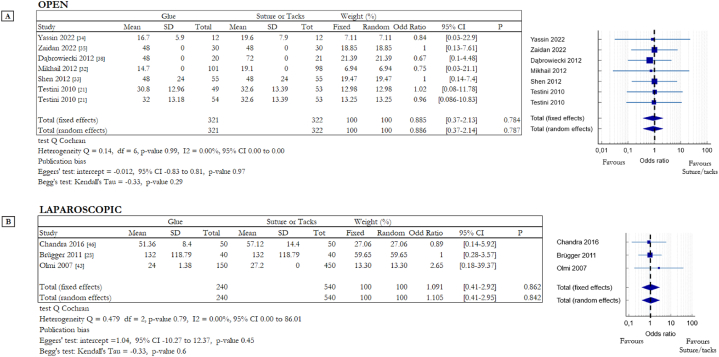
Summary data and forest plot comparing hospitalization time in hours following glue fixation versus suture or tack fixation of mesh during abdominal hernia repair procedures. The Mean column includes the mean values of hospitalization time, instead the SD column includes the standard deviation of these values. Odd ratios are charted with 95% confidence intervals. **A** Data obtained from open surgery (OS). **B** Data obtained from laparoscopic surgery (LS).

Seven studies evaluated the hospitalization time after abdominal hernia repair in OS and, in particular, six RCTs compared suture and cyanoacrylate-based glue [21,[32], [33], [34], [35],^38^] and Testini et al. analysed suture vs fibrin glue and also suture vs cyanoacrylate-based glue [21]. Instead in LS two RCTs evaluated the differences between fibrin glue and tacks [43,46] and only one compared cyanoacrylate-based glue with tacks [25]. The Forest plots in Fig. 8 compare the data; statistical analysis shows that there are no statistically significant differences about hospitalization time between glue and invasive mesh fixation techniques in OS (Fig. 8A) and LS (Fig. 8B). Data related to hospitalization time in OS gave the following results: OR 0.88, 95% CI 0.37 to 2.14, p-value 0.78 (random effects model). There was no heterogeneity among the studies (Q = 0.14, df = 6, p = 0.99, I2 = 0 %). For LS the results are: OR 1.1, 95% CI 0.41 to 2.95, p-value 0.86 (random effects model). There was no heterogeneity among the studies (Q = 0.48, df = 2, p = 0.79, I2 = 0 %).

### 3.5 Return to work

**Fig. 9:**
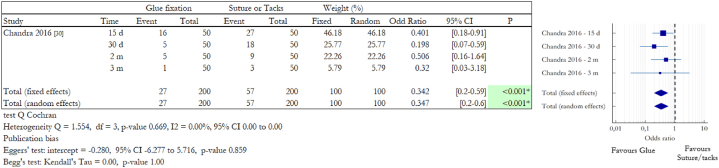
Summary data and forest plot comparing the number of patients unable to return to work following glue fixation versus suture or tack fixation of mesh during abdominal hernia repair procedures. In the Time column d stands for days and m stands for months. Event represents the number of patients that have not been able to return to work after surgery out of the total number of patients involved in the study. Odd ratios are charted with 95% confidence intervals.

Another aspect investigated is the ability of patients to return to their normal activities at 15, 30, 60 and 90 days after surgery. A special emphasis was placed on assessing how many patients returned to work or not and they were thus able to live a normal life. Chandra et al. evaluated how many people were not able to return to work because of pain or discomfort after laparoscopic procedures and the results of the study are reported in Fig. 9 [46]. The selected RCT evaluated the use of tacks and fibrin glue as mesh fixation system. Data show greater difficulty in returning to normal life in patients treated with tacks instead of glue. 15 days after surgery, 16 patients out of a total of 50 treated with glue were unable to return to normal life, but in the suture group, the number was significantly higher, as many as 27 patients out of 50 were unable to return to work and consequently to a normal life (32% vs 54%, p = 0.026*). After 30 days, the differences between the two groups increased. Only 5 patients in the glue group were unable to return to work while there were still 18 patients in the suture group unable to return to a normal life (10% vs 36%, p = 0.002*). Two months after surgery, there were still 5 people who did not return to work in the glue group, and 9 in the suture group (10% vs 18%, p = 0.249). After three months, they were reduced to 1 and 3 (2% vs 6%, p = 0.307) respectively.

The overall comparison of the data indicates that there is a statistically significant difference between the groups with data that favour the glue one (OR 0.347, 95% CI 0.2 to 0.6, p-value <0.001*, random effects model). There was no heterogeneity among the studies (Q = 1.554, df = 3, p = 0.669, I2 = 0 %).

This outcome was also analysed in RCTs on OS, but no sufficient and comparable numerical data were obtained to conduct a statistical analysis. Sözen et al. [37] compared the duration of incapacity to work between patients who were treated with fibrin glue and patients treated with suture. Data obtained from this study reveal a statistically significant difference between the two groups; on average, patients treated with surgical glue were unable to return to work for 5 days compared to 8 days for patients treated with suture (p value < 0.001*). The same study also shows the different complete healing time, 8.13 ± 7.88 days on average for the glue group versus 12.08 ± 8.59 for the suture group. Testini et al. also evaluate the mean time to return to work for three different group and they obtained: 20.4 days in the suture group, 20.3 days in the fibrin glue group, 19.8 d in the N-butyl-2-cyanoacrylate group with a p value of 0.6 [21].

## 4 Discussion

This meta-analysis compared the different mesh fixation techniques used in inguinal hernia repair procedures, according to different parameters, such as recurrence and occurrence of early and chronic pain in the postoperative periods (as primary outcomes) and duration of surgery, patient hospitalization time, seroma and hematoma formation, and the ability of patients to return to work (as secondary outcomes). According to the International Guidelines for Groin Hernia Management, mesh-based hernia repair is the standard treatment for adult symptomatic hernia [4]. There are several mesh fixation techniques including suture, fibrin glues, cyanoacrylate-based glues, absorbable or metallic tacks, self-gripping meshes, in addition there are also non-fixation techniques, and some postoperative outcomes are dependent on these different approaches. The present analysis compared invasive (suture/tacks) and non-invasive (glue-based) mesh fixation techniques, in terms of both primary and secondary outcomes, with distinction between the surgical procedure adopted. In view of the results of this meta-analysis and the features of the included papers, the use of cyanoacrylate-based or fibrin glues reduces early and significantly reduces chronic pain. Our results show a reduction in post-operative pain with the use of surgical glues, however, it can be seen that, in open surgery, there is no statistically significant difference between glues and sutures/tacks. On the other hand, the data are statistically significant in laparoscopic surgery where, the use of an invasive or non-invasive fixation device seems to have a major impact on the perception of post-operative pain (Fig. 2). The same result was obtained in other meta-analysis. Colvin et al. [41] evaluated acute early pain but only in open inguinal hernia repair, and it was shown that the mean VAS score postoperatively varied among the studies from 2 to 5 cm. Using the random effects model, the severity of early pain was reduced with glue fixation (MD = -7.92, 95 % CI -13.17 to −2.66, Z = 2.95, p = 0.003). Habib Bedwani et al. [42] evaluated the same aspect only in laparoscopic inguinal hernia repair and they obtained that VAS scores on day 1 were lower for glue fixation (mean difference −0.55 cm (95% CI –1.27 to 0.17); p = 0.13), but with significant heterogeneity (I^2^ = 96%).

Chronic pain is another important outcome which has a significant impact on the quality of life of patients. Comparing the data, a statistically significant difference between invasive and non-invasive mesh fixation systems is evident. It results that, in open surgery, only 19 of 729 patients of the glue group suffer chronic pain, instead in the suture/tacks group 58 of 721 patients have the same discomfort (2.6% vs 8.04%). In laparoscopic surgery, data are also in favour of the glue group, only 8 of 370 patients of the glue group suffer chronic pain, in contrast with suture/tacks group where the 52 of 676 patients have the same discomfort (2.16% vs 7.70%). This aspect is closely related to the results obtained for early pain; less early pain reduces the risk of developing chronic pain. This outcome was evaluated in several reviews; for example, Antoniou et al. [47] evaluated the incidence of chronic pain but only in laparoscopic procedures of inguinal hernia repair. They obtained that chronic pain was reported in 6.2% and 11.8% of patients in glue or mechanical fixation groups, respectively (OR 0.46, 95% CI 0.22 to 0.93), with no heterogeneity across studies (I^2^ = 0%), demonstrating a better result with glue. Phoa et al. [15] obtained that the incidence of chronic pain was 9.29% following glue fixation and 8.62% following sutures fixation, without any significant difference between the suture group and glue group (OR 1.10, 95% CI 0.73, 1.65; p = 0.65). The incidence of chronic pain depends on mesh fixation technique, but it seems to be multifactorial, with other important variables such as the mesh type and its pore size, as well as the operation procedure.

The rate of recurrence involves about 14% of patients undergoing prosthetic repair and it is mainly caused by displacement, slippage, or rupture of the mesh [48]. For this reason, it is important to understand how to ensure successful mesh fixation and to evaluate the differences between several techniques. It is, however, difficult to evaluate the impact of mesh fixation technique on the incidence of recurrences. The occurrence of recurrences usually appears years after surgery, while most RCTs evaluate follow-up periods of 12 or 24 months, which are not sufficient to obtain significant data on this problem. Analysing the data from different RCTs, we obtained results that are slightly in favour of surgical glues but not in a statistically significant way. Alabi et al. [3] in an overview of systematic reviews published in 2022, evaluated the same aspect and no significant differences were observed between different fixation techniques. Overall, most reviews showed no significant differences in recurrence rates between different mesh fixation methods, but it is important to consider that these studies assessed the incidence of recurrence after a short follow-up period of 6–12 months therefore the obtained results must be considered inadequate to draw definitive conclusions on the comparative effectiveness of glue and mechanical fixation [47,49]. In our meta-analysis, only two RCTs evaluated a follow up period of more than 24 months so the conclusions on this aspect must be considered with their limitations. The analysis gave the following results, recurrence occurred in 10 of 757 patients of the glue group for OS, instead in the suture/tacks group 18 of 736 patients have recurrence problems (1.32% vs 2.45%). Data from LS show that recurrence occurred in 2 of 286 patients of the glue group, instead in the suture/tacks group 8 of 589 patients have recurrence problems (0.7% vs 1.36%). Data are slightly in favour of surgical glues but not in a statistically significant way. The finding that glue has the same impact as penetrative means on the occurrence of recurrence is an interesting result. In fact, it permits to overcome the idea that only invasive means can provide stable fixation capable of preventing mesh displacement or detachment, events that can induce recurrence.

As secondary outcomes the occurrence of seroma and hematoma in relation to different prosthesis fixation methods were also evaluated. Seroma formation in OS appeared in the 5.57% of patients of glue group versus the 6.7% patients of sutures or tacks group without a statistically significant difference between the groups; in LS seroma formation appeared in the 3.37% in the glue group and in the suture/tacks the % was 8.3. The same result was obtained in the meta-analysis performed by Antoniou et al. (OR 0.85, 95% CI 0.35 to 2.06) [47] and by Colvin et al. (RR 0.91, 95 % CI 0.58–1.43; Z = 0.41, p = 0.68) [41]. Hematoma formation instead, is influenced by mesh fixation systems. Suture and tacks penetrate tissues even up to 7 mm resulting in tears and vessel damages. An injured vessel can cause bleeding and the possible hematoma formation. On the contrary, cyanoacrylate-based or fibrin glues are non-invasive medical devices, and their use is not associated with vessel damages. Our meta-analysis evaluated this aspect and data confirmed that the use of glues has a significant less impact on the occurrence of hematomas and bleeding, only 2.88% of patients in the glue group had hematoma formation in contrast with 10.7% in the suture/tacks group in OS (Fig. 6A), also in LS there is a statistically significant difference between glue and suture/tack groups (0.3% vs 3.18%). Several studies in literature show that damages to vessels and organs may occur not only during the mesh fixation but also later. For example, when a tack dislodges, followed by fascial disruption, it can penetrate deeper into the tissue and cause fascial defects and organ lacerations that may result in haemorrhaging [50].

Operation and hospitalization time are additional interesting outcomes, they are aspects linked not only with the mesh fixation methods but also with the surgical procedure (open or laparoscopic), with the skill and experience of the surgeon and with the characteristics of the hernia defect (dimension, position). For these reasons, it is not easy to compare and analyse data related to operative and post operative time. In our work we identified comparable data, and we obtained the forest plots (Fig. 7 for operation time: 7A open surgery, 7B laparoscopic surgery; Fig. 8 for hospitalization time: 8A open surgery, 8B laparoscopic surgery) that show no differences between invasive and non-invasive mesh fixation systems.

A useful parameter to describe patient wellness after surgery is the number of patients able to return to normal occupations at a certain timepoint after surgery. Among the glue group, early return of the patients to work was more frequent, possibly because there was less pain thus allowing lower limitations to normal work ability. The return to work at 30 days in the suture/tack group was only 64 % versus 90% of the glue group (p = 0.002*). Three months after abdominal surgery only one person in the glue group was unable to return to work while in the suture/tacks group 3. The overall comparison of the data indicates a statistically significant difference between the groups with data that favour the glue (Fig. 9).

Overall, the present meta-analysis examined aspects of interest to surgeons involved in repairing abdominal hernial defects. It is also of interest to evaluate the differences in cost between non-invasive and invasive fixation systems; clinical experts estimate the cost of tacking devices, complete with tacks, at roughly 206 GBP (range 160–241 GBP) instead a box of 12 sutures costs around to 23 GBP and 92 GBP. Fibrin glue is estimated to cost between 150 and 300 per procedure, instead a box of 10 cyanoacrylate glues has a total cost of ∼900 GBP [51]. In our meta-analysis, a statistical study was not conducted to assess cost-effectiveness but, in different studies, the impact of different fixation systems on health care costs was compared. Techapongsatorn et al. [52] made an economical evaluation of different mesh fixation systems in open an laparoscopic hernia repair. The research evaluated patient-specific data on the costs of hernia treatment, considering both hospital and societal perspectives. Hospital costs included room cost, operative, anaesthetic, laboratory, medical, and imaging costs. Social costs included direct nonmedical costs (transportation, extra meals) and indirect costs (lost productivity of the patient and caregivers, calculated by lost days multiplied by wages). From the analysis it was found that, in laparoscopic surgery, the inguinal hernia repair using self-gripping mesh has the highest total cost, followed by laparoscopic inguinal hernia repair using tacks and a better result was obtained with glue fixation. Open surgery has a lower cost impact with open inguinal hernia repair using glue representing the least costly alternative [52]. Panda et al. [53] also made a cost comparison between fibrin glue and tacks but only in laparoscopic procedures. In the following study, costs related to surgical time (600 GBP glue, 700 GBP tack), hospitalization time (400 GBP/day glue, 440 GBP/day tacks), seroma occurrence (3.60 GBP glue vs 8.64 GBP tacks) and neuralgia occurrence (0 GBP glue, 19.44 GBP tacks) were compared for a single surgical procedure. Important aspects such as complications associated with chronic pain and, of course, the costs of the materials used to fix the prosthesis were considered in the cost analysis. Overall, choosing glue for fixation instead of tacks results in a direct cost savings of 249 GBP per case. The literature shows that the use of tissue adhesives, even from an economic point of view, could be considered a valid alternative to the use of traditional penetrative fixation systems. Recent studies are evaluating the relation between the occurrence of chronic pain and healthcare costs, and, prosthetic fixation systems that result in less pain, such as surgical glues, may promote lower costs for healthcare and patients too. In our work it was not possible to make a statistical study on cost-effectiveness of various mesh fixation techniques and also the impact of different meshes and the patient quality of life as the number of studies existing at the moment, relative to these aspects, is insufficient to perform a significant meta-analysis. Other problems encountered in conducting this meta-analysis concerned the definition and assessment of chronic pain which presented varying degrees of differences among trials.

## 5 Conclusion

Abdominal hernia, and in particular inguinal hernia, is a very common pathological manifestation and the most reliable and safe solution to repair this defect is still debated. The use of tissue adhesive as mesh fixation device can represent a viable alternative to the use of invasive fixation methods, such as suture or tacks. The results of this meta-analysis show that synthetic or biological glues reduce early and, in a significant way, chronic pain without changing the rate of recurrence.

The incidence of seroma formation is not influenced by the mesh fixation technique while hematoma incidence is significantly lower in the not invasive mesh fastening glue group. Duration of surgery and hospitalization time are similar for glue and suture/tacks treated patients while the ability of patients to return to work is significantly better in those treated with glue. In conclusion, inguinal hernia repair by mesh fixation with cyanoacrylate-based glue or fibrin glue can be used as a simple not invasive, effective, and safe alternative to standard fixation with suture or tacks. Tissue adhesives seem to be able to improve postoperative rehabilitation of patients and to ensure a faster return to normal life.

## Human ethics and consent to participate declarations

Not applicable.

## Funding declaration

The authors did not receive support from any organization for the submitted work.

## Data availability statement

The authors declare that no data associated with our study has been deposited into a publicly available repository. Data included in article/supp. material/referenced in article. All the data that support the findings of this study are available from the corresponding author upon request.

## CRediT authorship contribution statement

**Cristiana Giordano:** Writing – review & editing, Writing – original draft, Investigation, Formal analysis, Data curation. **Elisabetta Rosellini:** Writing – review & editing, Supervision, Formal analysis, Data curation, Conceptualization. **Maria Grazia Cascone:** Writing – review & editing, Supervision, Formal analysis, Data curation, Conceptualization. **Francesca Di Puccio:** Writing – review & editing, Supervision, Formal analysis, Data curation, Conceptualization.

## Declaration of competing interest

The authors declare that they have no known competing financial interests or personal relationships that could have appeared to influence the work reported in this paper.
