# Supplementary material for: In vivo comparison of mesh fixation solutions in open and laparoscopic procedures for inguinal hernia repair: A meta-analysis
Source: Heliyon. 2024 Mar 23;10(7):e28711. doi: 10.1016/j.heliyon.2024.e28711 (PMC11059548; doi:10.1016/j.heliyon.2024.e28711)
Supplement: Multimedia component 1 [file mmc1.docx]

# Supplementary Material

# Bias assessment

## Primary outcomes - Open Surgery


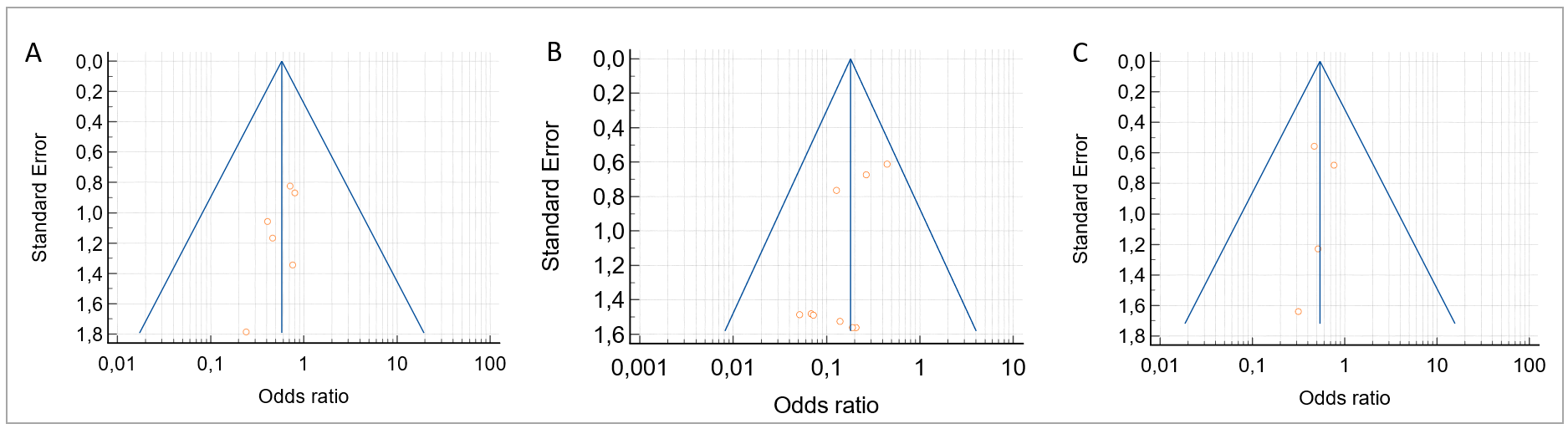


**Figure 1** Funnel plots. (**A**) Funnel plot for the outcome measure VAS score. (**B**) Funnel plot for the outcome measure chronic pain. (**C**) Funnel plot for the outcome measure recurrence

## Primary outcomes - Laparoscopic Surgery


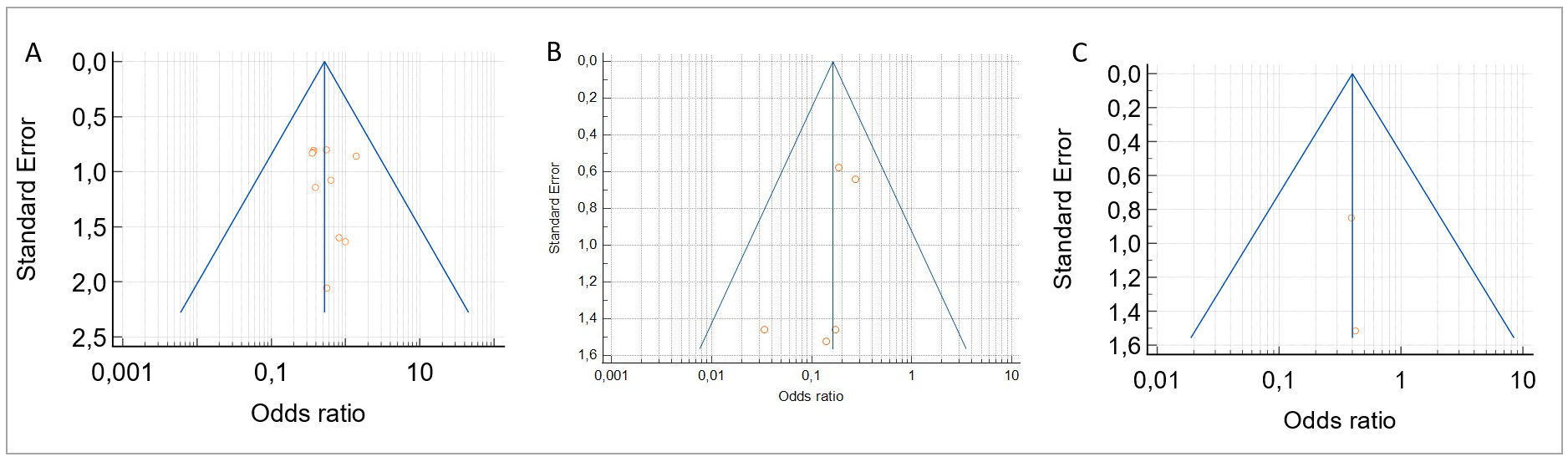


**Figure 2** Funnel plots. (**A**) Funnel plot for the outcome measure VAS score. (**B**) Funnel plot for the outcome measure chronic pain. (**C**) Funnel plot for the outcome measure recurrence

## Secondary outcomes - Open Surgery


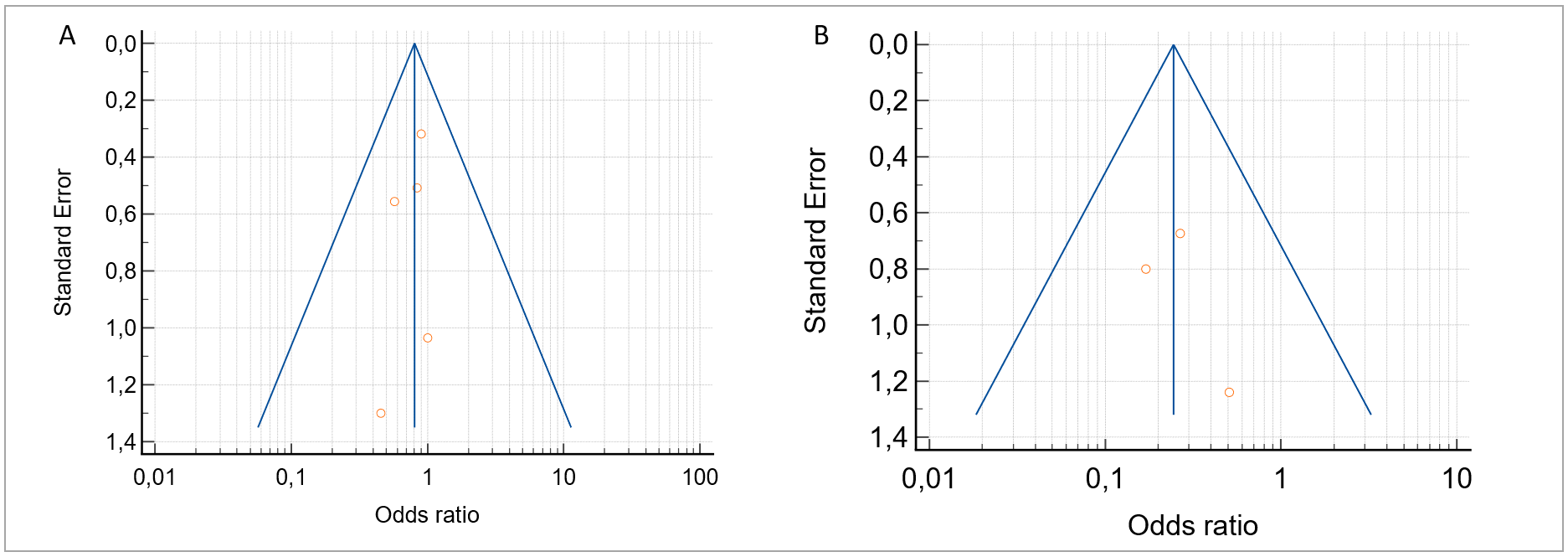


**Figure 3** Funnel plots. (**A**) Funnel plot for the outcome measure seroma. (**B**) Funnel plot for the outcome measure hematoma

## Secondary outcomes - Laparoscopic Surgery


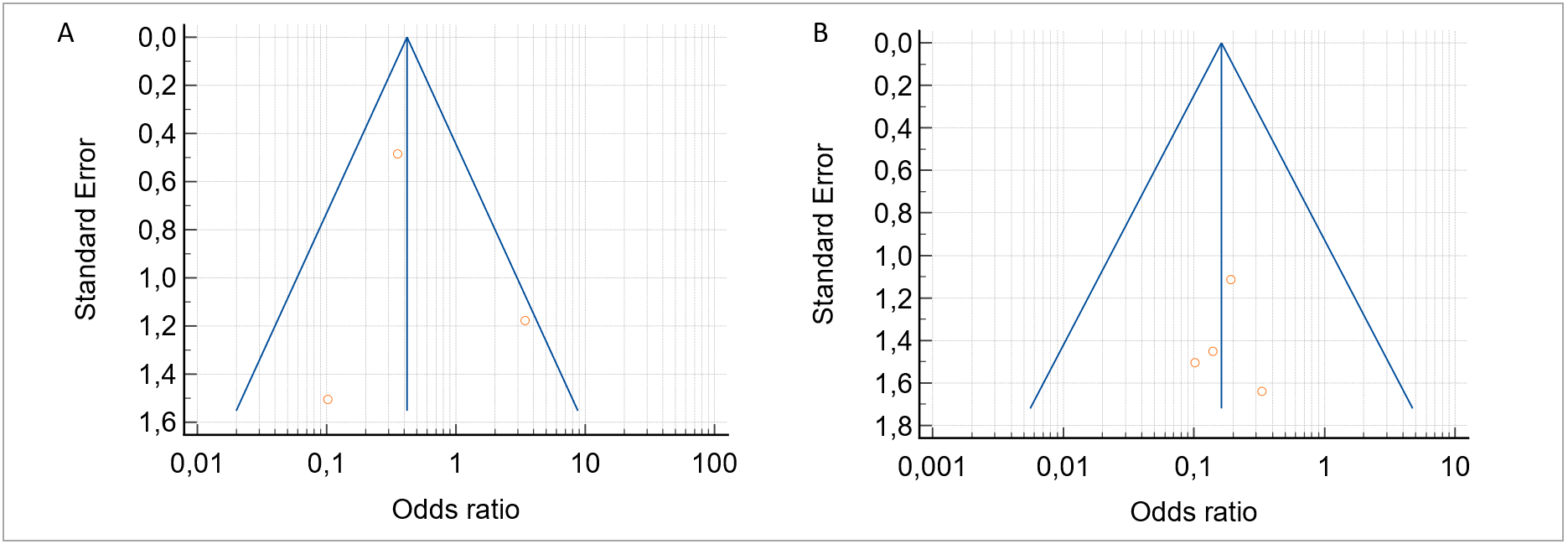


**Figure 4** Funnel plots. (**A**) Funnel plot for the outcome measure seroma. (**B**) Funnel plot for the outcome measure hematoma

## Additional outcomes - Open Surgery


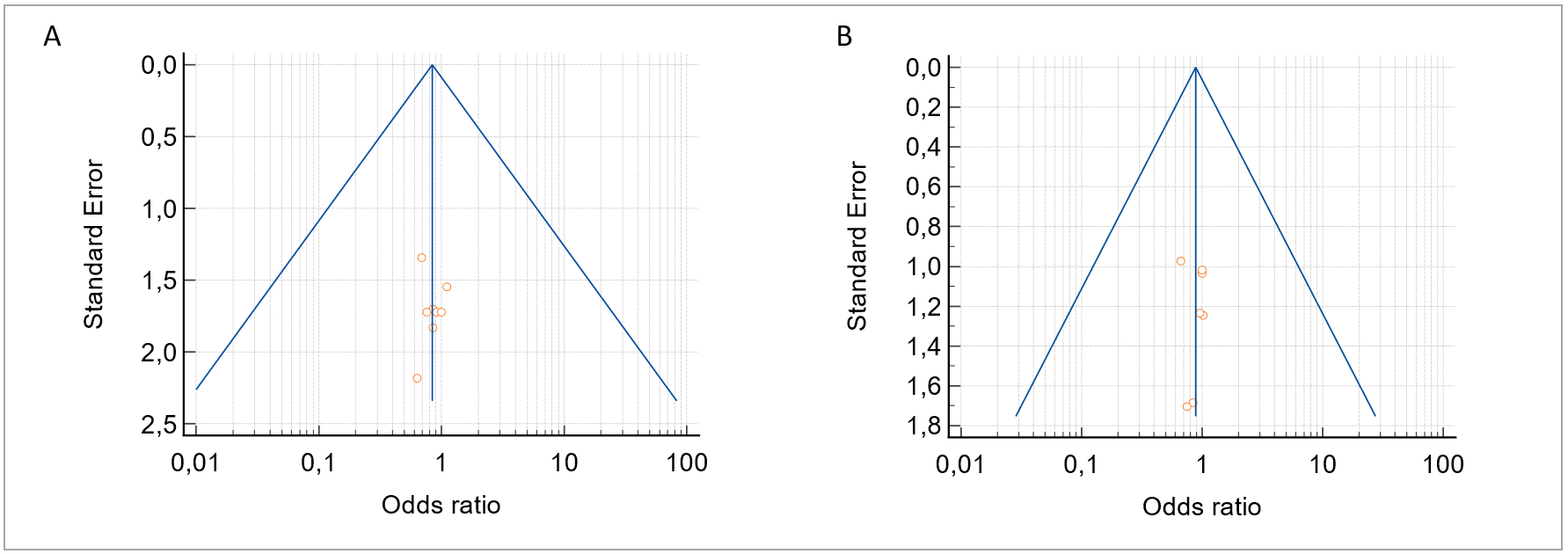


**Figure 5** Funnel plots. (**A**) Funnel plot for the outcome measure operative time. (**B**) Funnel plot for the outcome measure hospitalization time

## Additional outcomes – Laparoscopic Surgery


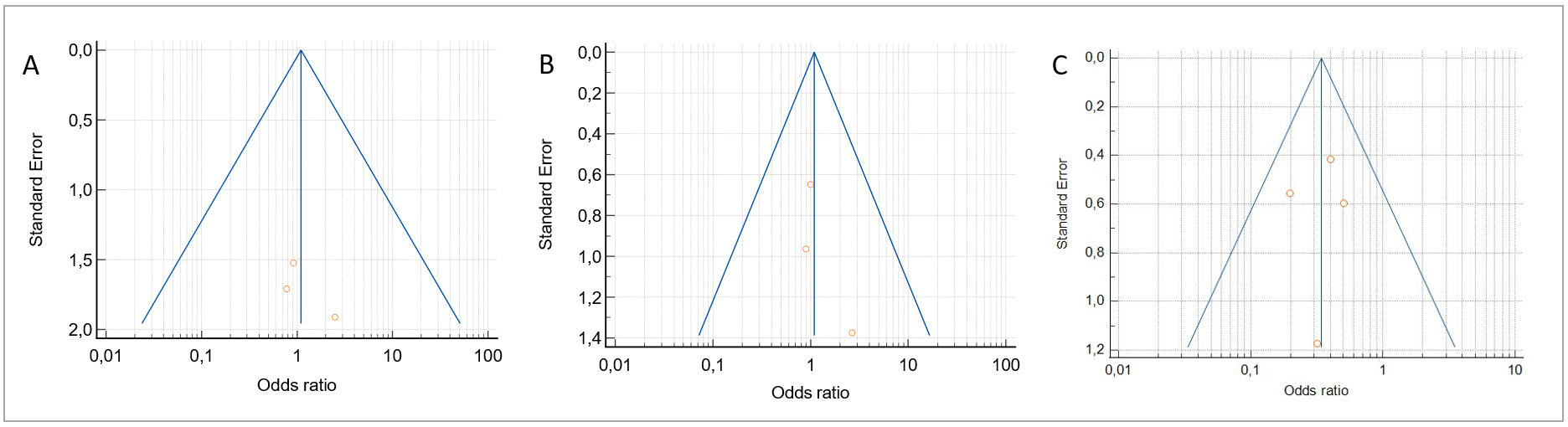


**Figure 6** Funnel plots. (**A**) Funnel plot for the outcome measure operative time. (**B**) Funnel plot for the outcome measure hospitalization time. (**C**) Funnel plot for the outcome measure return to work
